# Osteolipoma of the hand: A case report

**DOI:** 10.1016/j.ijscr.2024.110534

**Published:** 2024-10-28

**Authors:** Muhamad Naseh Sajadi Budi, Anglita Yantisetiasti, Ahmad Fitrah, Hans Kristian Handoko, Daniel Wirawan, Imam Ramdhani Abdurrahman

**Affiliations:** a Department of Orthopaedics and Traumatology, Oncology Division, Faculty of Medicine, Universitas Padjadjaran, Bandung, West Java, Indonesia; b Department of Radiology, Faculty of Medicine, Universitas Padjadjaran, Bandung, West Java, Indonesia; c Department of Pathology Anatomy, Faculty of Medicine, Universitas Padjadjaran, Bandung, West Java, Indonesia

**Keywords:** Osteolipoma, Hand tumor, Benign tumor, Case report, Surgical excision

## Abstract

**Introduction:**

Osteolipoma is a rare variant of lipoma characterized by osseous metaplasia within adipose tissue. Its occurrence in the hand is exceptionally uncommon. This article aimed to report a case of osteolipoma in the hand.

**Case presentation:**

A 46-year-old right-handed Asian woman presented to the orthopedic polyclinic complaining of a painless mass on the palm of her left hand for four years. Physical examination revealed a solid, mobile, non-tender mass with well-defined margins. Radiographic imaging showed a well-circumscribed soft tissue mass with fat and central calcification. The patient underwent surgical excision, and a histopathological examination confirmed the osteolipoma. A six-month follow-up revealed no recurrence, and the patient could resume her daily activities.

**Discussion:**

This case highlights the importance of including osteolipoma in the differential diagnosis of hand masses, especially those with radiographic evidence of calcification. The rarity of this entity in the hand requires a high index of suspicion for accurate preoperative diagnosis.

**Conclusion:**

While rare, osteolipoma of the hand should be considered in the differential diagnosis of soft tissue masses with osseous components. Complete surgical excision remains the treatment of choice, offering diagnostic confirmation and therapeutic intervention.

## Highlights

• Osteolipoma, a rare variant of lipoma with osseous metaplasia, was diagnosed in the hand of a 46-year-old woman
• Radiographic imaging revealed a well-circumscribed soft tissue mass with fat and central calcification, leading to surgical excision
• Osteolipoma is managed with surgical excision and histopathological examination to exclude malignancy
• This case emphasizes the importance of including osteolipoma in the differential diagnosis of hand masses with calcification, despite its rarity in this location

## 1 Introduction

Osteolipoma is a rare variant of lipoma, characterized by the presence of mature osseous tissue within a lipomatous neoplasm. While lipomas are common benign soft tissue tumors, osteolipomas account for fewer than 1 % of all lipomas. These tumors can occur in various anatomical locations, but their presence in the hand is uncommon [1]. The etiology of osteolipomas remains unclear; they may arise from pluripotent mesenchymal cells or through metaplasia of fibroblasts into osteoblasts. Their slow-growing nature often results in a delayed diagnosis, as patients may remain asymptomatic for extended periods. Diagnosing osteolipomas can be challenging due to their rarity and similarity to other soft tissue masses. Imaging studies play a crucial role in preoperative assessment. However, a definitive diagnosis typically requires histopathological examination [[2], [3], [4], [5]].

Osteolipoma treatment generally involves surgical excision, which is usually curative. The prognosis is excellent, with a low recurrence rate. However, the unique location presents specific challenges related to preserving function and aesthetics [1,5,6]. The updated literature regarding hand osteolipomas was minimal, as it is a rare diagnosis. This article aimed to report a case of osteolipoma of the hand per the Surgical Case Report (SCARE) 2023 guidelines criteria [7].

## 2 Case illustration

A 46-year-old right-handed Asian woman presented to the orthopedic surgery polyclinic complaining of a painless mass on the palm of her left hand for four years. The lump was initially the size of a marble and grew to the size of a ball within two years. She had no lumps anywhere else and denied a history of malignancy, reduced body weight, and decreased appetite. The family history of malignancy was unclear. She was working as a housewife. There was a history of trauma last year, and a plate was installed on the left forearm. No history of allergies, congenital diseases, chronic diseases, or family members having similar conditions existed.

**Fig. 1:**
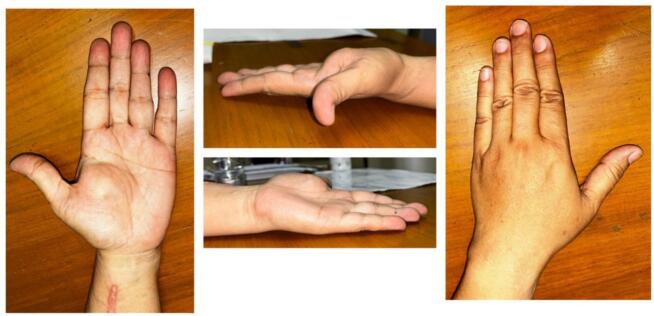
Osteolipoma of the hand.

**Fig. 2:**
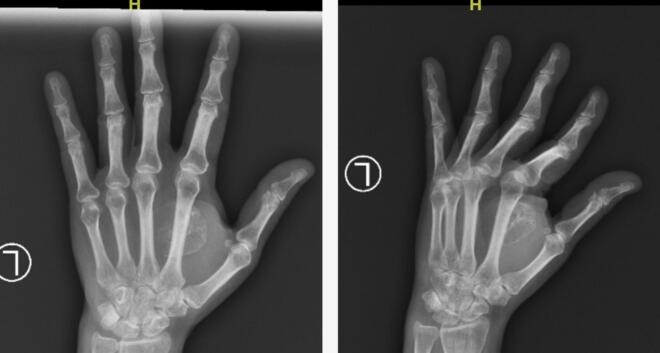
Anteroposterior and lateral view of hand X-ray showing a soft tissue mass with internal calcifications.

**Fig. 3:**
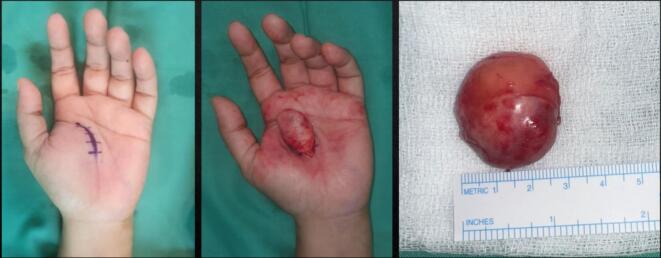
Intraoperative image of surgical excision showing a 2.8 cm osteolipoma.

**Fig. 4:**
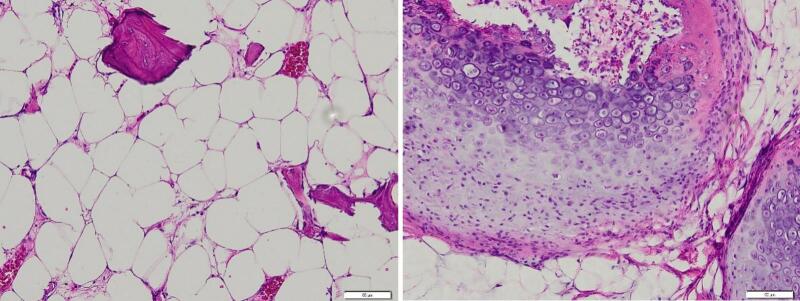
Histopathological examination showing mass composed of mature fat cells of various sizes. The edges appear covered with a connective tissue capsule and osteo-chondroid matrix.

**Fig. 5:**
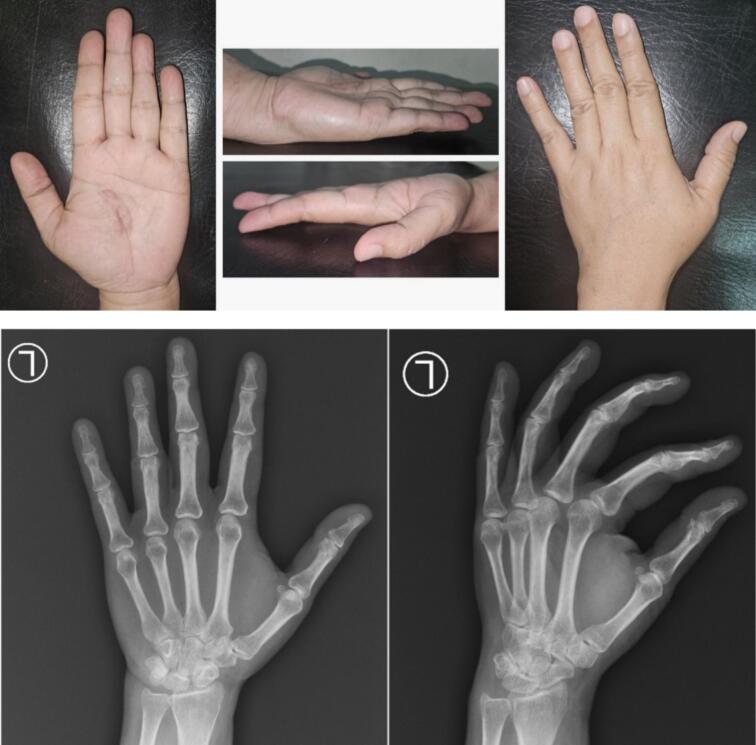
Clinical image and x-ray one month following surgical excision of osteolipoma.

**Fig. 6:**
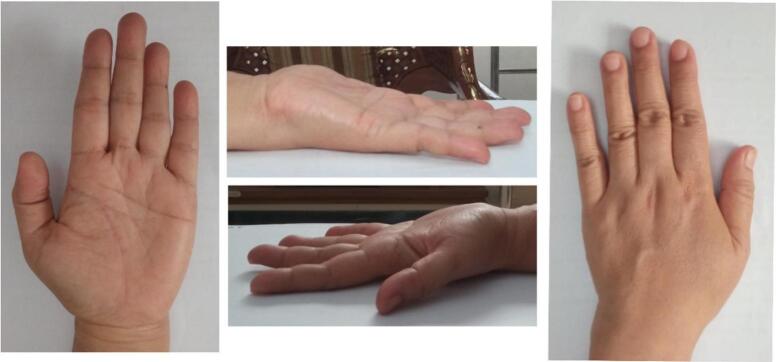
Clinical image six months following surgical excision of osteolipoma.

**Fig. 7:**
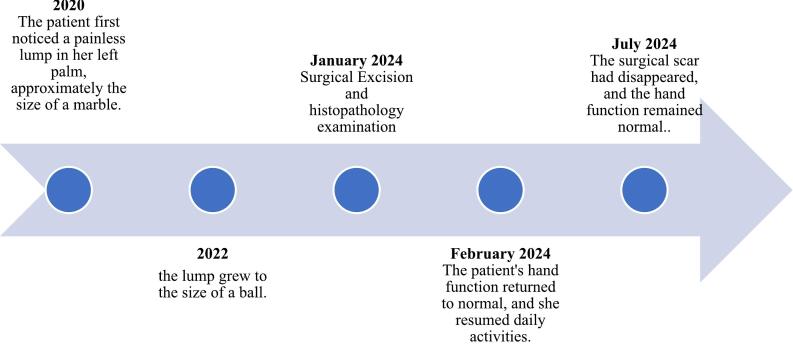
Timeline of the case.

Physical examination revealed a solid, non-tender, mobile mass sized 2 × 1.5 × 1 cm with a well-defined margin. The distal sensibility was normal compared to the other side. The range of motion was within the standard limit (Fig. 1). Anteroposterior and lateral view of the hand X-ray showed a mass with fat and internal calcifications (Fig. 2). We then performed a surgical excision with general anesthesia and histopathological examination, which revealed a brownish-white lump, size 3 × 2.8 × 2.3 cm, with a soft texture (Fig. 3). The lamellation was hard and brownish-yellow. Microscopic examination showed a mass composed of mature fat cells of various sizes, which grow hyperplastic, firm, and grouped. The nucleus was on the border. Lymphocyte cells invaded the connective tissue septa, accompanied by bleeding. This is often indicative of blood vessel rupture or leakage in the area. The edges appeared covered with a connective tissue capsule and osteo-chondroid matrix (Fig. 4). The surgery was uneventful, and the pain was minimal. The patient thought that the surgery process was tolerable for her and the post-operative pain was minimal. The patient was discharged from hospital on the day of surgery. A one-month follow-up revealed that the hand function was normal (Fig. 5). The patient was able to resume her daily activities, and she was satisfied with the result of the surgery. After six months of follow-up, the scar disappeared, and the hand function was still normal. There was no recurrence of the lesion (Fig. 6). The timeline of the case was presented in Fig. 7.

## 3 Discussion

Osteolipoma is a rare variant of lipoma characterized by the presence of mature bone within a lipomatous neoplasm. It accounts for fewer than 1 % of all lipomas, while lipomas have been estimated to afflict 1 % of the population, with an incidence of 2.1 per 1000 people per year [1,8]. The head and neck are the most common sites of reported osteolipomas. The majority of lipomas include osseous tissue related to bone (either inside or next to it). Osteolipoma independent of bone tissue has been observed in a few reports [9]. The exact epidemiology of osteolipoma of the hand is unknown, as it is primarily documented through case reports [4].

The exact etiology of osteolipomas remains unclear. Two main theories are: 1) they may arise from pluripotent mesenchymal cells capable of multidirectional differentiation, and 2) they could result from metaplasia of fibroblasts into osteoblasts within a pre-existing lipoma, perhaps due to insufficient nourishment within the lipoma mass [10]. Osteolipoma can occur due to numerous extrinsic causes, such as mechanical stress, recurrent trauma, chronic inflammation, or ischemia. Our case supports the notion that chronic irritation or mechanical stress might play a role in osteolipoma formation, given the tumor's location on the palm, an area subject to frequent minor trauma when using the hand [11]. In our case, the patient is a housewife who often works by hand.

Diagnosing osteolipoma of the hand involves several unique challenges, primarily due to its rarity and the resemblance it shares with other soft tissue masses. These tumors are not commonly encountered, and their presentations can vary significantly, which complicates the diagnostic process. Furthermore, the symptoms associated with osteolipomas, such as a palpable mass, pain, or restricted mobility, are common to many other types of soft tissue tumors, making clinical diagnosis based solely on symptoms and physical examination challenging [3]. The approach to the differential diagnosis in cases suspected of osteolipoma includes considering a variety of possible conditions. For instance, osteochondroma, a bone growth capped by cartilage, can appear similar but is typically identifiable by its continuity with the underlying bone [12]. Calcifying aponeurotic fibroma is more common in younger individuals [13], Meanwhile, giant cell tumors of the tendon sheath are smaller and localized [14]. Another differential to rule out is myositis ossificans, characterized by bone formation within muscle following trauma. It has a distinct etiology and appearance in imaging [15,16].

Imaging plays a crucial role in narrowing down the differential diagnosis. Plain radiographs are the first-line diagnostic imaging of osteolipoma, revealing the presence of calcification. MRI and CT scans may help delineate the lipomatous nature of the lesion. However, definitive diagnosis relies on histopathological examination. Osteolipoma is histologically described as mature adipose tissue with a multifocal region of bone tissue [4,5,17]. We only perform X-rays, not MRIs or CT scans, considering the cost-benefit. In our case, the X-ray image showed internal calcification, which was suspicious for osteolipoma.

The management of osteolipomas typically involves surgical excision, which serves both diagnostic and therapeutic purposes. Given that these tumors are well-encapsulated, complete removal is usually straightforward and carries a low risk of recurrence while preserving the hand's function and appearance. While alternative methods like liposuction might be considered, they pose a higher risk of incomplete tumor removal and potential damage to nearby nerves or vessels. [4] In our case, complete excision was achieved without compromising hand function and cosmetic appearance.

The choice of anesthesia in the surgical removal of a hand osteolipoma depends largely on the size and location of the tumor, as well as the patient's overall health and preference. Local anesthesia is commonly preferred for smaller, well-defined lesions in the hand, as it allows for a quick recovery and discharge process. For larger or more complex lesions, regional anesthesia or even general anesthesia might be necessary to ensure patient comfort and allow for a more extensive exploration and excision [18]. We used general anesthesia in our case at the patient's request.

The surgical approach to removing an osteolipoma in the hand typically involves a careful incision designed to minimize damage to the surrounding tissues. The incision is strategically placed to provide optimal access to the tumor while preserving the cosmetic appearance and functional integrity of the hand. After the incision, dissection is carried out around the mass, taking care to preserve the vital structures such as nerves and blood vessels. Osteolipomas, being well-encapsulated, generally allow for a clean dissection as they are often separate from the surrounding soft tissues. The tumor is then carefully mobilized and excised in its entirety to ensure complete removal and reduce the risk of recurrence. The encapsulated nature of osteolipomas typically facilitates this process, as the capsule can often be used as a plane for dissection, ensuring that the lesion is removed with minimal residual tissue [4,19].

During the operation, meticulous attention is paid to achieving hemostasis to prevent postoperative complications such as hematoma formation. The intraoperative assessment often involves ensuring that the tumor has been completely excised, which might include the use of intraoperative imaging techniques like ultrasound or X-ray if the preoperative imaging suggests deeper or more extensive bone involvement. The excised tissue is typically sent for histopathological examination to confirm the diagnosis of osteolipoma by identifying the characteristic mature adipose tissue and bone within the lesion [4,5,20].

Following the tumor excision, the surgical site is carefully closed in layers to promote optimal healing and aesthetic outcomes. Sutures are typically placed in such a way that they minimize scarring and preserve hand function. Postoperative care involves monitoring for signs of infection, managing pain, and ensuring that the patient regains full function through rehabilitation exercises. Careful postoperative follow-up is essential to ensure there is no recurrence and that hand functionality is fully restored. Comparing this case to others reported in the literature helps reinforce the understanding of osteolipoma's typical presentations and outcomes, guiding future clinical approaches to similar cases [4,19]. Panev et al. reported osteolipoma size 5.2 cm × 3 cm in the first dorsal web space that limited the thumb's range of motion [4]. Demark et al. reported osteolipoma 2.5 cm × 1.7 cm × 1.6 cm in the left wrist. Chen et al. reported osteolipoma 3.6 × 5 × 3.6 cm in right thenar eminence. Teoh et al. reported an osteolipoma that was painful during activity. Our case was osteolipoma 3 × 2.8 × 2.3 cm in the palm. Other reported locations were the knee, thigh, palate, oral cavity, salivary gland, head, and neck. All cases were resolved with simple surgical excision, and no recurrence occurred after a 6 months until 3 years of follow up [1,4,[9], [10], [11],^18^,[20], [21], [22]].

## 4 Conclusion

While rare, osteolipoma of the hand should be considered in the differential diagnosis of soft tissue masses with osseous components. Complete surgical excision remains the treatment of choice, offering diagnostic confirmation and therapeutic intervention.

## Consent

The patient has been explained the procedures and possible risks of the surgery and given written informed consent. My manuscript does not contain any data on individuals. The patient provided written informed consent for publication and any accompanying images.

## Ethical approval

The ethical committee of our university provided ethical approval for this study on May 20th, 2024. The patient provided written informed consent to publish this case report and accompanying images.

## Funding

This research received no specific grant from funding agencies in the public, commercial, or not-for-profit sectors.

## Author contribution

Muhamad Naseh Sajadi Budi: Oncology Orthopaedic Surgeon(Operator), Conceptualization, Visualization, Methodology, Writing and Supervision.

Anglita Yantisetiasti: Pathology anatomy interpreting, Conceptualization, Visualization, Methodology, Writing and Supervision.

Ahmad Fitrah: Radiology interpreting, Conceptualization, Visualization, Methodology, Writing and Supervision.

Hans Kristian Handoko, Daniel Wirawan, Imam Ramdhani Abdurrahman: Writing, operation assistant.

## Guarantor

The guarantor in this study is Muhammad Naseh Sajadi Budi Irawan.

## Declaration of competing interest

The authors declare that there is no conflict of interest regarding the publication of this paper.
